# Non‐Invasive Estimation of Systemic Drug Exposure Using Exhaled Breath Metabolomics in Humans: A Translational Proof‐of‐Concept Study

**DOI:** 10.1002/cpt.70437

**Published:** 2026-08-07

**Authors:** Kai Fricke, Diego M. Baur, Kapil Dev Singh, Felix Schmidt, Kathrin Müsch, Noriane A. Sievi, Marco Di Donato, Anton Schmick, Jonas Herth, Silvia Ulrich, Pablo Sinues, Marian Galovic, Malcolm Kohler

**Affiliations:** ^1^ Department of Pulmonology University Hospital Zurich Zurich Switzerland; ^2^ Faculty of Medicine University of Zurich Zurich Switzerland; ^3^ University Children's Hospital Basel Basel Switzerland; ^4^ Department of Biomedical Engineering University of Basel Basel Switzerland; ^5^ Department of Neurology University Hospital Zurich Zurich Switzerland

## Abstract

Accurate assessment of systemic drug exposure in humans remains challenging, as conventional approaches rely on intermittent blood sampling and laboratory‐based assays that are invasive and provide limited temporal resolution. Exhaled breath contains volatile and semi‐volatile compounds arising from drug metabolism and downstream biological processes, suggesting that it may serve as a non‐invasive matrix for integrated pharmacokinetic sensing. We conducted a prospective observational study using exhaled breath metabolomics based on secondary electrospray ionization high‐resolution mass spectrometry (SESI‐HRMS) to estimate systemic drug exposure from a breath‐sampling session. A total of 117 adults provided 127 paired breath–serum measurements under routine outpatient conditions. Model performance was evaluated using repeated k‐fold cross‐validation and quantified using concordance correlation coefficients. Breath‐derived estimates of valproic acid, levetiracetam, lamotrigine, and lacosamide showed moderate concordance with measured serum concentrations, with concordance correlation coefficients ranging from 0.733 to 0.895 across medications. The lacosamide findings should be considered preliminary because of the limited subgroup size (*n* = 16). The observed concordance supports proof‐of‐concept exposure estimation but does not establish clinical interchangeability with serum‐based therapeutic drug monitoring. The previously defined 11‐feature VPA breath signature retained predictive utility in a separate adult cohort, supporting its cross‐cohort transferability within the present analytical framework. These findings support the feasibility of exhaled breath analysis for non‐invasive estimation of systemic drug exposure across pharmacologically distinct medications. Breath analysis should currently be considered complementary to serum‐based monitoring, and external multicenter and longitudinal validation is required before clinical implementation.

**Study Highlights**

**WHAT IS THE CURRENT KNOWLEDGE ON THE TOPIC?**

Therapeutic drug monitoring of antiseizure medications commonly relies on venous blood sampling and laboratory assays that are invasive, intermittent, and typically drug‐specific. Exhaled breath metabolomics has emerged as a potential non‐invasive approach for pharmacological monitoring, but evidence in adults and across multiple medications remains limited.

**WHAT QUESTION DID THIS STUDY ADDRESS?**

Can SESI‐HRMS applied to exhaled breath estimate systemic exposure to commonly used antiseizure medications in adults under routine clinical conditions, and does this approach extend beyond valproic acid to pharmacologically distinct compounds?

**WHAT DOES THIS STUDY ADD TO OUR KNOWLEDGE?**

Breath‐based models showed moderate analytical agreement with measured serum concentrations for valproic acid, levetiracetam, lamotrigine, and lacosamide. Previously reported valproic acid‐associated breath features were independently reproduced in an adult cohort, supporting reproducibility of breath‐derived pharmacokinetic signatures. These findings suggest that a single breath sample may enable multi‐drug exposure estimation without blood sampling.

**HOW MIGHT THIS CHANGE CLINICAL PHARMACOLOGY OR TRANSLATIONAL SCIENCE?**

Exhaled breath analysis may represent a low‐burden complementary modality for pharmacokinetic monitoring, particularly where repeated sampling, rapid assessment, or multi‐drug estimation is desirable. With further external validation, this approach could broaden access to therapeutic monitoring and support future development of precision dosing strategies.

Epilepsy affects more than 50 million people worldwide and typically requires long‐term treatment with antiseizure medications (ASMs).^1^ Achieving sustained seizure control while minimizing adverse effects remains challenging due to substantial interindividual variability in ASM metabolism and pharmacokinetics.^2^ Therapeutic drug monitoring (TDM) is therefore central to clinical management, supporting dose individualization, adherence assessment, and prevention of toxicity.^3^ However, conventional TDM relies on intermittent plasma or serum sampling, an invasive, resource‐intensive approach that provides only brief snapshots of drug exposure. These limitations can delay recognition of subtherapeutic levels, emerging toxicity, or treatment nonresponse, contributing to therapeutic decisions that are often reactive rather than anticipatory.^4^ More broadly, scalable and minimally invasive strategies for estimating systemic drug exposure remain limited, highlighting an unmet need for accessible and patient‐friendly monitoring approaches.

Non‐invasive exhaled breath analysis has gained attention as a potential complementary approach for TDM.^5^ Despite growing interest, breath‐based approaches to TDM have not been established for routine clinical practice. This reflects a combination of technical and infrastructural challenges, including the need for standardized breath sampling protocols, robust quality control procedures, access to high‐resolution analytical instrumentation, and validation across diverse clinical settings. In addition, breath metabolomics has historically been applied primarily in exploratory, single‐center studies, limiting opportunities for large‐scale replication. Addressing these challenges requires methodologically rigorous human studies that demonstrate analytical reproducibility, biological plausibility, and potential generalizability across drugs and patient populations.

Advances in secondary electrospray ionization high‐resolution mass spectrometry (SESI‐HRMS) enable direct detection of volatile and semi‐volatile compounds, including parent drugs and metabolites, from exhaled air without sample preparation.^6^ Breath‐derived metabolic signatures may also reflect downstream pharmacodynamic processes, providing a potentially integrated measure of drug exposure and biological effect with minimal patient burden.^7^ Together, these properties position breath as a dynamic sensing medium rather than a passive surrogate of circulating drug levels.

Initial feasibility data support this concept. In a predominantly pediatric cohort, Singh and colleagues reported moderate to strong concordance between SESI‐HRMS breath profiles and serum concentrations of total and estimated free valproic acid (VPA).^8^ However, the generalizability of these findings to adult populations and to ASMs with diverse pharmacokinetic properties remains unknown, particularly in the context of polytherapy.

To address these gaps, we conducted an observational study in adults with epilepsy to evaluate the performance and translational potential of breath‐based drug exposure estimation using SESI‐HRMS. Our aims were (i) to evaluate and extend breath‐derived predictive models for total and estimated free VPA concentrations, and (ii) to assess whether this approach extends to three commonly used ASMs: levetiracetam (LEV), lamotrigine (LTG), and lacosamide (LCM). By assessing multiple ASMs from a single breath‐sampling session within a routine clinical setting, this study explores the potential generalizability and current limitations of breath analysis as a complementary modality for TDM in adults.

## METHODS

### Study design and participants

We conducted a single‐center observational study at the University Hospital Zurich between September 2022 and August 2024. Adults aged 18 years or older with a clinically confirmed diagnosis of epilepsy who were receiving stable doses of one or more ASMs were eligible for inclusion. All participants were in pharmacokinetic steady state at the time of sampling. Patients were recruited during routine outpatient visits at the USZ Epilepsy Centre.

A total of 117 patients (mean age 43.6 ± 17.2 years; 50 [43%] female) contributed 127 valid breath measurements. Ten patients provided repeat samples at follow‐up visits. Of 134 attempted recordings, seven were excluded due to technical issues or missing matched serum ASM measurements (**Table** **S1**). Epilepsy etiology was classified according to International League Against Epilepsy (ILAE) criteria.

The study was approved by the Cantonal Ethics Committee of Zurich (BASEC No. 2019‐00030), conducted in accordance with the Declaration of Helsinki and Good Clinical Practice guidelines, and registered at ClinicalTrials.gov (NCT05456009). All participants provided written informed consent prior to participation.

### Serum antiseizure medication measurements

Venous blood samples were processed and analyzed by the accredited clinical chemistry laboratory of University Hospital Zurich according to validated standard operating procedures. Blood samples were collected in serum separator tubes, allowed to clot at room temperature, and centrifuged within 2 hours of collection; serum aliquots were analyzed immediately or stored at −20°C until measurement. Total VPA concentrations were quantified using a homogeneous enzyme immunoassay (HEIA). Serum concentrations of LEV, LTG, and LCM were measured using validated liquid chromatography–tandem mass spectrometry (LC–MS/MS) assays routinely employed in clinical practice. All assays were performed with appropriate calibrators and quality control materials, and analytical performance was monitored in accordance with laboratory accreditation standards. Estimated free VPA reference concentrations were calculated from measured serum total VPA and albumin using the correction described by Parent *et al*.,^9^ reflecting common clinical practice where direct measurement of unbound VPA is not routinely available. A separate breath‐based regression model was trained directly against these formula‐derived estimated free VPA reference concentrations; the albumin‐based equation was not applied to breath‐predicted total VPA values.

### Instrumentation

#### Real‐time breath analysis platform

Real‐time breath analysis was performed using a standardized analytical platform based on SESI‐HRMS, as previously described with modifications. The platform consisted of three main components: (i) an exhalation interface for monitoring and guiding the breathing maneuver (Exhalion, Fossil Ion Technology, FIT, Spain), (ii) a secondary electrospray ionization source optimized for breath analysis (Super SESI, FIT, Spain), and (iii) a high‐resolution Orbitrap mass spectrometer (Q Exactive Plus, Thermo Fisher Scientific, Germany).

At the beginning of each measurement day, analytical performance was verified by a system suitability assessment performed prior to breath data acquisition, as previously described.^10^ Platform stability was evaluated using a certified reference gas mixture comprising eight target compounds (Dalian Special Gases, Dalian, China). The mixture was delivered at a nominal concentration of 2 ppm and a total flow rate of 10 L min^−1^ using a calibrated gas dilution system (Model 2010, Sabio Environmental, Round Rock, USA). Breath measurements were initiated only after the system demonstrated acceptable performance according to predefined criteria.

Signals obtained from the reference gas were evaluated using a custom‐developed analysis tool implemented in MATLAB (version 2.2). Multivariate comparison against historical reference measurements was performed using Hotelling's *T* ^2^ statistics derived from principal component analysis (PCA). Acceptance of the system suitability test required that the multivariate score remained within the 95% confidence limits of previous measurements, that statistical process behavior complied with Nelson control rules, and that the response intensity for α‐terpinene exceeded 1 × 10^7^. In parallel, background ion patterns were automatically compared with historical profiles to identify potential contamination or carryover within the analytical system. Only breath data acquired following successful completion of this quality control procedure were included in subsequent analyses.

#### Exhalation maneuver monitoring and guidance

The Exhalion interface was designed to control and improve the reproducibility of the exhalation maneuver by providing real‐time feedback on key respiratory parameters, as described in detail previously.^11^ The system continuously measured carbon dioxide (CO_2_) concentration (%), pressure drop (mbar), exhalation flow rate (L min^−1^), and exhaled volume (L) during each breath.

Participants exhaled through a sterile, single‐use antibacterial and antiviral medical‐grade mouthpiece consisting of a commercially available spirometry filter (MicroGard™ II, Vyaire Medical, USA; inner diameter 3 cm; filtration efficiency 99.98% for bacteria and 99.92% for viruses). Downstream of the mouthpiece, the filter was connected to an autoclavable interface housing a calibrated flow restriction. The pressure drop across the restriction (range 0–20 mbar; accuracy 2.5%; precision 0.1 mbar) was used to calculate the exhalation flow rate (range 0–15 L min^−1^; accuracy 2.5%). Total exhaled volume was automatically estimated by detecting the onset of exhalation and integrating the flow rate over time.

Side‐stream capnography was used to monitor CO_2_ concentration (range 0–20%; accuracy 5% of the reading) at an approximate sampling flow rate of 0.5 L min^−1^. Absolute pressure was also measured to correct CO_2_ and flow‐rate readings for barometric pressure variations.

#### Data acquisition and system architecture

All respiratory parameters were acquired at a sampling frequency of 1.5 Hz and stored as text files for subsequent analysis. The Exhalion system incorporated a main module containing a touchscreen interface, micro‐computer, integrated sensors, and dedicated firmware enabling fully autonomous operation. Automated routines for sensor calibration were embedded within the firmware.

The main module and flow‐restriction interface were connected via two 1/8″ outer‐diameter tubes for CO_2_ and pressure measurements. Nafion tubing was used to prevent condensation. The dead volume of the side‐stream tubing and sensors was < 5 cm^3^, resulting in a maximum CO_2_ signal delay of approximately 0.5 s. The total dead volume of the system was dominated by the mouthpiece filter, as the Exhalion interface was designed to minimize additional dead volume. The Exhalion device was connected downstream to the Super SESI ionization source.

#### Secondary electrospray ionization

The Super SESI source was optimized for real‐time analysis of exhaled breath metabolites. A controlled fraction of the exhaled breath was continuously directed into the ionization chamber. Ionization was achieved using a nano‐electrospray of 0.1% formic acid in water.

The electrospray was generated using a 20 μm inner‐diameter TaperTip silica capillary emitter (New Objective, USA). The solution was driven through the emitter at a pressure of 0.8 bar, resulting in a stable nano‐electrospray with typical steady‐state spray currents of approximately 120 nA, as monitored by a nanoamperemeter.

The sampling line temperature was maintained at 130°C, and the ion‐source core temperature was set to 90°C. Both the sampling line and ionization chamber core were silica‐coated to minimize analyte adsorption onto internal surfaces.

#### Gas flow control and breath transfer

When no breath sample was introduced, the ionizer was continuously swept with clean nitrogen filtered through an integrated activated charcoal filter. The nitrogen flow was set to exceed the intake flow of the mass spectrometer by 0.4 L min^−1^. An exhaust mass flow controller was set to 0.7 L min^−1^, ensuring that the fraction of breath entering the ionizer was fixed at 0.3 L min^−1^, independent of fluctuations in exhalation pressure.

The dead volume of the sampling line and ionizer was approximately 10 cm^3^, corresponding to a transit time of ~2 s for exhaled breath to reach and fully flush the ionization chamber.

#### High‐resolution mass spectrometry

The Super SESI source was directly coupled to a Q Exactive Plus Orbitrap mass spectrometer and operated as an electrospray ionization (ESI) source. The MS source parameters were as follows: sheath gas flow rate 60, auxiliary gas flow rate 2, spray voltage 2.80 kV, capillary temperature 275°C, and S‐lens RF level 55.0.

All real‐time breath measurements were performed in full MS mode unless otherwise specified. Spectra were acquired over an m/z range of 70–1000 at a resolution of 140,000 (at m/z 200), using two microscans per spectrum, an automatic gain control (AGC) target of 1 × 10^6^, and a maximum injection time of 500 ms. Instrument control and data acquisition were performed using Q Exactive Tune software (version 2.12).

#### Mass spectrometer calibration

External calibration of the mass spectrometer was performed monthly using a Pierce™ FlexMix calibration solution (Thermo Fisher Scientific). Internal calibration was achieved by enabling lock masses corresponding to common background contaminants.

For positive ion mode, the following lock masses were used: m/z 59.04914, 101.09609, 129.12739, 136.02155, 143.10666, 169.12231, 279.15909, 371.10123, 445.12002, and 519.13882. For negative ion mode, lock masses included m/z 59.01385, 73.02950, 89.02442, 101.06080, 115.07645, 143.10775, 157.12340, 255.23295, and 283.26425.

### Breath sampling protocol

Breath analysis using SESI‐HRMS was performed immediately after venous blood collection. Serum ASM concentrations were determined using standard clinical chemistry assays at the University Hospital Zurich (**Table** **S2**). Sampling was performed during routine outpatient visits and was not standardized to trough conditions; consequently, samples were obtained at variable intervals after the most recent ASM dose, and the exact dose‐to‐sampling interval was not systematically recorded. Only end‐tidal scans acquired when exhaled CO_2_ exceeded 3% were retained for analysis, thereby reducing contributions from upper‐airway dead‐space air.

All breath measurements were conducted in the same clinical room under standardized environmental conditions, with ambient temperature maintained at 20°C. Participants were examined in the fasting state and were instructed to refrain from eating, drinking (except water), smoking, chewing gum, alcohol, tobacco, caffeine, or brushing their teeth for at least 1 hour prior to breath sampling.

Participants exhaled repeatedly through a sterile, single‐use spirometry filter (MicroGard, Vyaire Medical, USA) into a heated transfer line connected directly to the SESI‐HRMS system. The transfer line consisted of a 50 cm stainless steel tube (4 mm inner diameter) coated with Silconert to minimize analyte adsorption and was connected to the auxiliary (AUX) curtain gas port of the Orbitrap mass spectrometer. To prevent water condensation and reduce compound losses on the tubing walls, the transfer line was maintained at 130°C and thermally insulated using heating tape.

A digital capnograph continuously monitored exhaled volume and CO_2_ concentration to identify alveolar (end‐tidal) breath in real time. Each participant performed 12 controlled exhalations in total, corresponding to approximately 9–12 L of exhaled air, acquired across six reproducible exhalations per ionization mode. Measurements were performed twice per participant, once in positive ion mode and once in negative ion mode, enabling reproducible acquisition of breath‐derived molecular profiles while the analytical system continuously monitored the exhaled air.

### Data processing

Raw mass spectra (Thermo Fisher .raw files) were pre‐processed using a proprietary pipeline (Deep Breath Intelligence AG, Switzerland), implemented via in‐house C# console applications built on Thermo Fisher Scientific's RawFileReader and MATLAB. Following acquisition, end‐tidal exhalation windows were defined from the Exhalion capnography trace (e.g., CO_2_ > 3%) to ensure that only the end‐tidal fraction contributed to downstream analyses. Scans within exhalation windows were averaged to generate one representative averaged centroid spectrum per measurement, followed by removal of satellite peaks and recalibration. Finally, the averaged centroid spectra from different measurements were binned within ±2 ppm using MATLAB's ksdensity function. In total, 19,790 mass spectral features were obtained (16,265 in positive ion mode and 3525 in negative ion mode). Signal intensities were log_10_‐transformed. Features with more than 50% zero values were excluded. Remaining zero values were treated as left‐censored and imputed using Bayesian lognormal modeling with Markov chain Monte Carlo sampling (PyMC v5.22.0; ArviZ v0.21.0). Model convergence was assessed using effective sample size and Gelman–Rubin statistics (R̂).

### Feature annotation and metabolite assignment

Breath‐derived molecular features were defined as reproducible m/z signals detected by SESI‐HRMS in positive and negative ionization modes. Since SESI‐HRMS captures a composite breath metabolomic profile, endogenous and drug‐derived signals were not physically separated before modeling. Instead, medication‐associated features were identified through supervised feature selection within each training fold according to their relationship with the corresponding serum drug concentration. Putative metabolite annotations were assigned by accurate‐mass matching against curated metabolite databases, taking into account elemental composition, ionization mode, and known biochemical transformations. Metabolite identities should be interpreted as putative according to Metabolomics Standards Initiative level 2–3 (MSI criteria) rather than confirmed structures. Where multiple candidate annotations were possible for a given m/z feature, all plausible assignments were retained. Biological interpretation therefore focuses on consistency with known metabolic pathways rather than definitive identification of individual compounds. The present study was designed to predict paired serum parent‐drug concentrations from composite breath profiles rather than to quantify individual metabolites. Because targeted reference measurements and authentic‐standard confirmation of individual breath metabolites were not performed, metabolite‐level analytical accuracy was not assessed.

To assess the cross‐cohort transferability of the previously defined VPA feature signature, all 11 VPA‐associated features identified in the earlier predominantly pediatric cohort were evaluated in the present dataset.^8^ Features were matched within ±2 ppm, required agreement in ionization mode, and were assessed for consistency in the direction of association with serum VPA concentration. All 11 features met these criteria and constituted the fixed predictor set for the adult‐cohort VPA prediction analyses. The corresponding previously reported and present‐cohort *m*/*z* values, mass differences, putative annotations, ionization modes, and association directions are provided in **Table** **S3**. This analysis evaluated transferability of the previously defined feature signature within the present analytical framework and did not constitute independent confirmation of molecular identity or reproducibility across laboratories, instruments, processing pipelines, or sites.

### Machine learning and statistical analysis

Separate regression models were developed to predict total VPA, estimated free VPA, LEV, LTG, and LCM concentrations from breath metabolomic data. Support vector machine regression (kernlab v0.9.33) and partial least squares regression (plsmod v1.0.0.9000, pls v2.8.5) were utilized for prediction. For the VPA prediction analyses, the previously defined 11‐feature signature was fixed before model fitting and was used as the predictor set throughout nested cross‐validation; adult‐cohort feature selection was therefore not used to redefine the VPA predictor set.

For the LEV, LTG, and LCM models, feature importance was evaluated within each training fold using four complementary algorithms: least absolute shrinkage and selection operator (LASSO; glmnet v4.1.10), Boruta (Boruta v9.0.0), minimum redundancy maximum relevance (mRMR; mRMRe v2.1.2.2), and recursive feature elimination (RFE; caret v7.0.1). Feature importance scores were normalized and aggregated to generate a consensus ranking to improve robustness across feature‐selection methods.

Model training employed nested cross‐validation, consisting of an outer 10‐fold loop with 10 independent resamplings and an inner 10‐fold cross‐validation for hyperparameter tuning using tune_race_anova (finetune v1.2.0). To prevent information leakage, all measurements from the same participant were assigned to the same cross‐validation fold. Model performance was summarized using concordance correlation coefficient (CCC), root mean squared error (RMSE), mean absolute error (MAE), coefficient of determination (*R* ^2^), and bias (yardstick v1.3.2). Samples lacking matched serum measurements were excluded from analysis.

### Threshold calibration for therapeutic classification

Continuous model predictions were categorized as within or outside the therapeutic range using established serum reference intervals: total VPA 350–700 μmol L^−1^, estimated free VPA 35–69 μmol L^−1^, LEV 59–235 μmol L^−1^, LCM 1–10 mg L^−1^, and LTG 11.7–58.6 μmol L^−1^. These thresholds correspond to accepted serum therapeutic ranges used in routine clinical TDM (**Table** **S2**). Classification performance was assessed using PPV, NPV, and AUC. These classification analyses are exploratory and dependent on cohort‐specific distribution of concentrations.

### Model interpretability and uncertainty quantification

For the LEV, LTG, and LCM models, feature‐importance rankings from LASSO, Boruta, mRMR, and RFE were combined to generate consensus sets of explanatory variables for model interpretation. Interpretation of the VPA models was based on the fixed, previously defined 11‐feature signature. Model uncertainty was quantified using 1000 bootstrap resamples. Median values and 95% confidence intervals were reported for CCC, AUC, PPV, NPV, RMSE, and MAE to characterize robustness and reproducibility. All analyses were performed using R (v4.5.0; tidymodels v1.4.0, parsnip v1.4.0, recipes v1.3.1, rsample v1.3.1, workflowsets v1.1.1) and Python (v3.9.21).

### Reproducibility, transparency, and access to analytical workflows

Feature alignment and peak matching were performed using fixed m/z tolerances defined a priori and did not depend on outcome variables or dataset‐wide data distributions. All subsequent preprocessing and modeling steps, including feature filtering, normalization, Bayesian imputation, and model training, were specified before model evaluation and performed within the training portion of each cross‐validation fold before being applied unchanged to the corresponding held‐out test data, thereby minimizing the risk of information leakage.

To support verification, **Table** **S3** provides the drug‐associated m/z features used for model training together with the cross‐cohort matching information for the 11 previously reported VPA‐associated features. While the proprietary implementation is available through licensing, the principal preprocessing and modeling steps are described to facilitate independent implementation and evaluation using alternative software.

## RESULTS

### Study cohort and paired breath‐serum measurements

One hundred and seventeen adults with epilepsy contributed 127 valid paired breath–serum measurements. Clinical and demographic characteristics by ASM are summarized in **Table** **1**. Breath samples covered predominantly therapeutic and subtherapeutic serum concentrations across multiple ASMs, enabling assessment of breath‐based exposure estimation within the observed concentration range under routine outpatient conditions. Supratherapeutic and toxic concentrations were sparsely represented. Most participants were receiving more than one ASM at the time of sampling.

**Table 1:** Study cohort characteristics, including antiseizure medication use, seizure types, and comorbidities

	Valproic acid	Lamotrigine	Levetiracetam	Lacosamide
	(*n* = 31)	(*n* = 72)	(*n* = 34)	(*n* = 16)
Age, years (SD)	39.2 (14.7)	44.3 (17.0)	47.9 (20.6)	47.6 (15.4)
Sex				
Female (%)	9 (29)	32 (44)	8 (24)	7 (44)
Male (%)	22 (71)	40 (56)	26 (76)	9 (56)
BMI (SD)	25.4 (4.8)	24.8 (4.0)	24.2 (3.9)	27.0 (5.9)
Etiology^a^				
Unknown	10	22	5	3
Genetics	15	12	5	4
Structural	6	36	22	9
Metabolic	0	0	0	0
Infectious	0	2	2	0
Number of ASMs				
1	16	42	19	0
2	9	24	12	11
3	3	5	3	3
4 or more	3	1	0	2

ASM, antiseizure medication; BMI, body mass index; ILAE, International League Against Epilepsy; SD, standard deviation.

^a^ According to ILAE classification.

### Prediction of VPA concentrations using breath analysis

**Figure 1:**
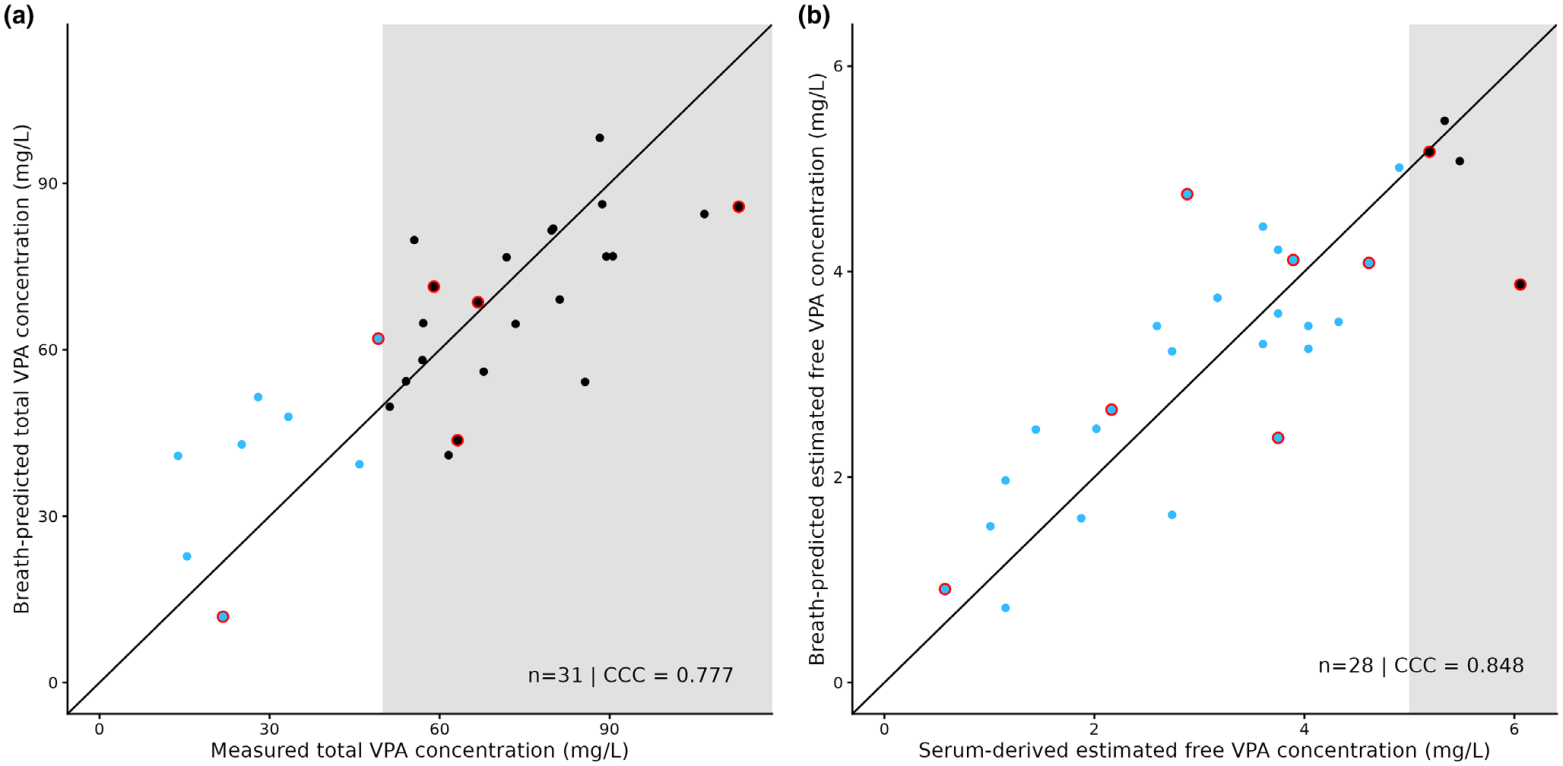
Estimation of total and estimated free valproic acid concentrations using breath analysis. **Panel (a)** displays agreement between breath‐predicted and measured serum total valproic acid concentrations, and **Panel (b)** displays agreement between breath‐predicted estimated free valproic acid concentrations and serum‐derived estimated free valproic acid reference concentrations calculated using an albumin‐based correction. Breath‐based predictions were generated using regression models trained on selected mass spectral features. This agreement between predicted and measured concentrations was evaluated using the concordance correlation coefficient (CCC). The light gray shaded area indicates the serum‐based therapeutic range. Blue dots represent serum values outside the therapeutic window, while black dots indicate values within the therapeutic range. Red circles highlight measurements from patients who reported ASM‐related side effects at the time of sampling. ASM, antiseizure medication; CCC, concordance correlation coefficient; *n*, number of paired breath‐serum measurements.

**Figure 2:**
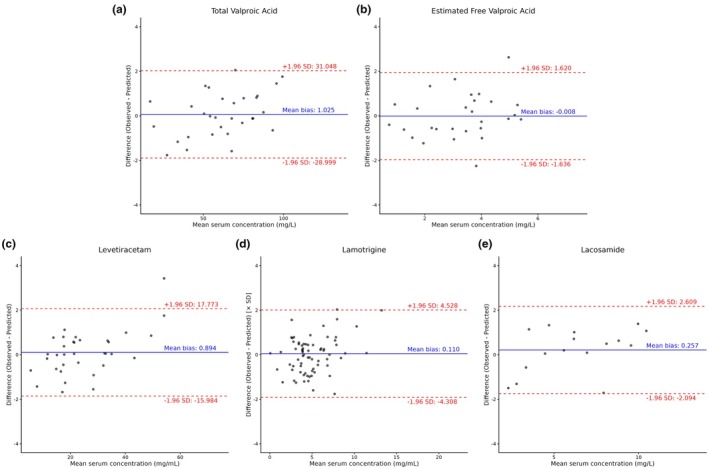
Bland–Altman agreement between breath‐predicted and measured serum antiseizure medication concentrations. Bland–Altman plots show the agreement between breath‐derived predictions and measured serum concentrations for (**a**) total valproic acid, (**b**) estimated free valproic acid, (**c**) lamotrigine, (**d**) levetiracetam, and (**e**) lacosamide. Each plot displays the difference between measured serum and breath‐predicted concentrations (observed – predicted) against the mean of the paired measured and predicted concentrations. The solid horizontal line represents the mean bias, and the dashed lines denote the 95% limits of agreement (LOA). Points above zero indicate underestimation by breath analysis, and points below zero indicate overestimation.

In the adult cohort, breath‐based predictions showed moderate to good agreement with measured serum concentrations, with CCCs of 0.777 for total VPA and 0.848 for estimated free VPA (**Figure** **1** **a**,**b**). All 11 VPA‐associated features identified in the earlier predominantly pediatric cohort^8^ were matched within ±2 ppm, occurred in the same ionization mode, and retained concordant directions of association with serum VPA concentration. The same 11‐feature signature constituted the fixed predictor set for the adult‐cohort VPA prediction analyses and retained predictive utility under nested cross‐validation (**Table** **S3**). The CCC was numerically higher for estimated free VPA than for total VPA. Predicted and measured concentrations showed an approximately linear relationship across the observed range. Most serum‐derived estimated free VPA reference concentrations were below the established therapeutic interval. When model outputs were categorized relative to established therapeutic reference intervals (**Table** **S2**), classification performance was influenced by cohort composition and class imbalance. In addition, agreement within 20% of serum values was observed in 53.3% of total and 63.6% of estimated free VPA predictions (**Table** **2**). Bland–Altman analysis demonstrated a mean bias close to zero for both total and estimated free VPA, with differences distributed around the bias line (**Figure** **2** **a,b**).

**Table 2:** Agreement between breath‐derived and serum antiseizure medication concentrations

	Total valproic acid	Estimated free valproic acid	Lamotrigine	Levetiracetam	Lacosamide
	(*n* = 31)	(*n* = 28)	(*n* = 72)	(*n* = 34)	(*n* = 16)
CCC (95% CI)	0.777 (0.627–0.867)	0.848 (0.654–0.952)	0.781 (0.664–0.877)	0.733 (0.562–0.820)	0.895 (0.799–0.936)
Bias (measured serum – breath‐predicted), mg/L	1.133	−0.082	0.914	0.116	0.232
95% LOA	−28.199 to 30.469	−1.579 to 1.413	−15.592 to 17.420	−4.452 to 4.686	−2.052 to 2.512
MAE	12.216	0.582	0.716	0.554	0.857
Within P20/P30, %	53.3% | 66.7%	63.6% | 72.7%	50.0% | 64.7%	34.5% | 45.0%	62.5% | 81.2%
In‐range agreement, %	83.8%	91.7%	88.2%	71.2%	100.0%
Therapeutic range	350–700 μmol/L	35–69 μmol/L	11.7–58.6 μmol/L	59–235 μmol/L	1–10 mg/L

Performance metrics are shown for total valproic acid, estimated free valproic acid, lamotrigine, levetiracetam, and lacosamide. Values include the concordance correlation coefficient (CCC) with 95% confidence interval, mean bias (measured serum − breath‐predicted), 95% limits of agreement (LOA), mean absolute error (MAE), percentage of predictions within 20% or 30% of the corresponding serum concentration (P20/P30), and agreement in classification relative to established serum therapeutic reference intervals. In‐range agreement denotes the proportion of breath‐derived estimates assigned to the same in‐range or out‐of‐range category as the paired serum measurement. In‐range agreement for lacosamide should be interpreted cautiously because all measured lacosamide concentrations were within the therapeutic reference interval. CI, confidence interval; CCC, concordance correlation coefficient; LOA, limits of agreement; MAE, mean absolute error; P20/P30, percentage of estimates within 20% or 30% of the corresponding serum concentration.

### Breath–based estimation of additional ASMs

**Figure 3:**
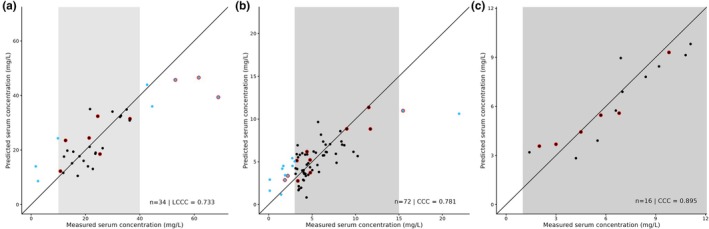
Breath‐based estimation of serum antiseizure medication concentrations. Scatterplots show the agreement between breath‐based predictions and measured serum concentrations for three commonly prescribed ASMs using breath analysis: **Panel (a)**, levetiracetam; **Panel (b)**, lamotrigine; **Panel (c)**, lacosamide. Breath‐based predictions were generated using regression models trained on mass spectral features. Each point represents an individual paired breath–serum measurement. The diagonal line indicates the line of identity (perfect agreement between predicted and measured values), and the light gray shaded area denotes the serum‐based therapeutic range. Blue dots represent values outside the therapeutic window, while black dots represent values within the therapeutic range. Red circles highlight measurements from patients who reported ASM‐related side effects at the time of sampling. The lacosamide findings should be considered preliminary because of the limited sample size. ASM, antiseizure medication; *n*, number of paired breath–serum measurements. [Correction added on 13 August 2026, after first online publication: Figure 3 has been updated in this version.]

To assess whether breath‐based exposure estimation can be applied beyond valproic acid, drug‐specific prediction models were developed for three additional ASMs with distinct pharmacokinetic properties. Models were trained for LEV (*n* = 34), LTG (*n* = 72), and LCM (*n* = 16) using drug‐associated breath features derived from SESI‐HRMS. LCM results should be interpreted as preliminary because of the limited sample size (*n* = 16; **Table** **S4**). Breath‐derived estimates demonstrated moderate analytical agreement across medications, with CCCs of 0.733 for LEV, 0.781 for LTG, and 0.895 for LCM (**Figure** **3** **a–c**).

Putative annotations for the most informative mass‐to‐charge (m/z) features contributing to each drug‐specific model are summarized in **Table** **S3**. These assignments are based on accurate‐mass matching and contextual biochemical interpretation and should be regarded as tentative rather than definitive molecular identifications in accordance with established metabolomics reporting standards.

When predictions were categorized relative to established therapeutic reference intervals, classification performance varied across medications and was constrained by class imbalance and limited representation of out‐of‐range concentrations. Detailed metrics are reported in **Table** **S4** and should be interpreted as exploratory and cohort‐dependent. Bland–Altman analyses for LEV, LTG, and LCM showed mean bias values close to zero, without clear evidence of systematic bias across the observed concentration ranges (**Figure** **2** **c–e**).

## DISCUSSION

This observational study demonstrates the feasibility of non‐invasive breath analysis using SESI‐HRMS to estimate systemic exposure to several commonly used ASMs, including VPA, LTG, LCM, and LEV. Importantly, these findings support the concept that exhaled breath can function as a potential integrated sensor of systemic drug exposure at the level of composite metabolic signatures arising from parent drugs and downstream metabolism rather than individual circulating analytes alone. TDM remains an important tool in epilepsy care, particularly when individuals experience breakthrough seizures, adverse effects, or require dose adjustments during polytherapy. However, conventional TDM relies on venepuncture, laboratory processing, and separate assays for each drug, which can be slow, invasive, and resource‐intensive.^2^ For certain ASMs, such as LEV and LCM, serum levels often correlate only modestly with clinical outcomes, highlighting the need for complementary approaches that capture pharmacological information beyond single‐point serum concentrations.^12^, ^13^ Breath analysis has theoretical advantages in this context: exhaled breath contains a mixture of volatile and semi‐volatile compounds originating from both parent drugs and downstream metabolism, thereby providing a more integrated chemical snapshot of systemic exposure.

The present findings suggest that SESI‐HRMS breath analysis can estimate ASM concentrations with moderate analytical agreement under the conditions studied, supporting its potential as a patient‐friendly adjunct to serum‐based monitoring. In clinical terms, concordance in the observed range suggests that breath‐derived estimates may help identify patients who warrant further assessment, but individual estimates should not yet be used as a stand‐alone basis for dose adjustment or medication changes. The study extends previous breath‐based estimation of VPA exposure by evaluating the transferability of the same 11‐feature signature originally identified in a predominantly pediatric cohort.^8^ All 11 features were matched within ±2 ppm, occurred in the same ionization mode, retained concordant directions of association with serum VPA concentration, and constituted the fixed predictor set used in the adult‐cohort VPA prediction analyses. These included features putatively compatible with established β‐ and ω‐oxidation products of VPA, such as 3‐heptanone and 4‐hydroxy‐γ‐lactone.^14^, ^15^ The retained predictive utility of this previously defined signature in a separate adult cohort supports its cross‐cohort transferability and strengthens its biological plausibility within the present analytical framework. However, these findings do not establish definitive molecular identity, which will require targeted structural confirmation or reproducibility across laboratories, instruments, operators, processing pipelines, or sites, and will require independent multicenter and cross‐platform validation.

The distinction between total and estimated free VPA is clinically important, because only the unbound fraction is pharmacologically active. In routine practice, free VPA is often inferred using albumin‐based equations rather than measured directly due to cost and logistical constraints.^16^ Estimated free valproic acid (VPA) concentrations in this study were derived using a widely applied albumin‐based correction,^9^ reflecting current clinical practice in settings where direct measurement of unbound VPA is not routinely performed. Accordingly, the serum‐derived reference concentrations and the corresponding breath‐predicted estimated free VPA concentrations should both be interpreted as estimates rather than direct measurements of the unbound fraction. Albumin‐based equations are known to introduce systematic uncertainty under certain physiological conditions, highlighting the limitations of formula‐based approaches to free drug estimation. The predominance of values below the estimated free VPA therapeutic interval may reflect the total VPA and albumin distributions represented in this cohort, but systematic underestimation by the albumin‐based correction cannot be excluded. In the absence of directly measured unbound VPA, the present study cannot distinguish between these explanations. The estimated free VPA analysis therefore evaluates whether breath metabolomic profiles can approximate a clinically used formula‐derived exposure metric, but it does not provide a protein‐binding‐independent measurement of unbound VPA. Prospective studies incorporating direct measurement of unbound VPA and correlation with clinical outcomes will be required to determine whether breath‐derived estimates provide clinically useful information beyond total VPA monitoring.

Beyond VPA, breath‐based exposure estimation was evaluated for three newer ASMs. Despite their comparatively low volatility, LEV, LTG, and LCM were all amenable to breath‐based prediction with CCC values between 0.733 and 0.895. Lower compound volatility may nevertheless increase susceptibility to incomplete transfer, surface adsorption, and variation in respiratory and airway conditions, thereby contributing to measurement variability. These results suggest that multi‐drug monitoring from a breath‐sampling session is feasible, supporting its potential to complement traditional workflows in which separate serum assays are required for each drug. This capability is particularly relevant for individuals receiving polytherapy, where even small pharmacokinetic shifts can alter tolerability or efficacy. Because a breath‐sampling session can provide exposure estimates for multiple drugs, breath analysis could support future approaches to polytherapy management by reducing the need for separate assays. Given class imbalance and modest subgroup sizes for some medications, classification metrics derived from therapeutic reference intervals should be interpreted as exploratory and cohort‐dependent rather than definitive indicators of clinical decision performance. In these settings, rapid, non‐invasive estimates may ultimately serve as screening information to identify patients warranting confirmatory serum testing.^17^ LCM results should be interpreted as preliminary because they were based on only 16 paired measurements. In a subgroup of this size, the comparatively high concordance may be sensitive to cohort composition and the represented concentration range and should not be interpreted as evidence of superior performance relative to the other ASMs.

A further strength of the study is the biological plausibility of the breath features contributing to the drug‐specific prediction models. These features were putatively annotated and interpreted in the context of known metabolic pathways for each medication, with biological interpretation based on consistency across features rather than definitive structural identification. Accordingly, these annotations should not be interpreted as validated quantitative measurements of individual metabolites. Establishing metabolite‐specific accuracy would require targeted analytical validation using confirmed compounds and appropriate reference measurements. For LEV, several associated m/z features were compatible with previously described phase I oxidative and phase II conjugative biotransformations, although direct structural confirmation was not performed. LTG‐associated features were similarly consistent with oxidative and glucuronidation‐related processes, while LCM‐associated signals aligned with reported oxidative pathways, including metabolites related to O‐desmethyl‐LCM.

The recurrence of oxygen‐ and nitrogen‐containing compounds across multiple models is consistent with shared oxidative pathways; however, because the predictive features were selected from composite breath profiles, the present analysis cannot definitively distinguish direct drug‐ or metabolite‐derived signals from endogenous metabolic responses associated with drug exposure. Differences in predictive performance across ASMs may reflect underlying pharmacokinetic characteristics, including the narrower dynamic range and stronger metabolic signal observed for estimated free VPA, as well as the broader concentration ranges and greater interindividual variability associated with LEV and LTG.

Alternative sampling media such as saliva, dried blood spots, and microneedle‐extracted interstitial fluid have been explored for ASM measurement, each with specific benefits and limitations.^18^, ^19^, ^20^ Breath sampling is distinctive in that it requires minimal consumables, causes no discomfort, and can be repeated with minimal burden. SESI‐HRMS offers the additional advantage of real‐time analysis, enabling the possibility of more dynamic pharmacokinetic assessment during clinical encounters.

**Figure 4:**
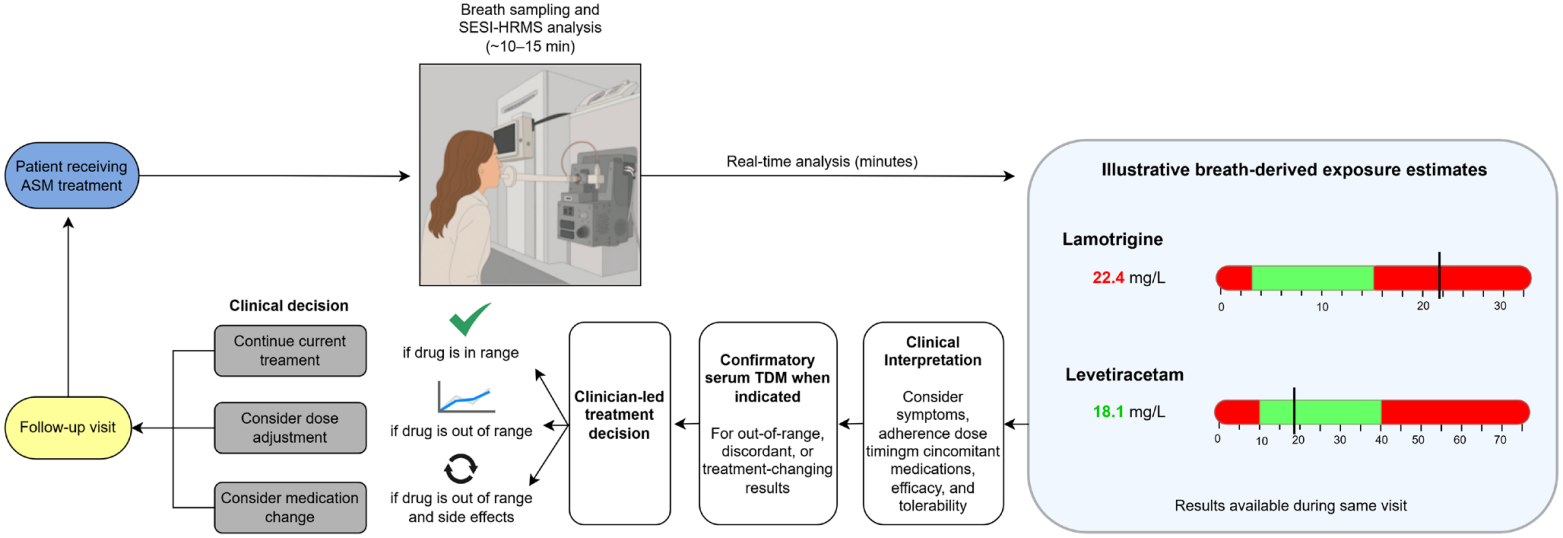
Proposed clinical integration of breath analysis for antiseizure medication management. This schematic illustrates how real‐time breath analysis using secondary electrospray ionization high‐resolution mass spectrometry could be integrated into routine care to support therapeutic drug monitoring of ASMs. A patient provides an on‐site breath sample, which is analyzed immediately to provide estimates of serum concentrations of multiple ASMs. In this example, predicted serum levels of lamotrigine (18.6 μg/mL) are above the illustrated therapeutic range, whereas the predicted lacosamide concentration (6.3 μg/mL) is within its therapeutic range. Response and side‐effect risk scores are shown as conceptual examples of additional decision‐relevant information. This multi‐analyte, non‐invasive approach may support therapeutic drug monitoring by providing non‐invasive estimates of systemic drug exposure and could complement conventional monitoring approaches. Clinical decisions would remain guided by standard clinical assessment and confirmatory serum testing, with breath analysis serving as a complementary source of pharmacokinetic information.

While this study does not assess pharmacokinetic curves, the online nature of SESI‐HRMS breath acquisition raises the possibility of future applications in dose optimization or acute care settings, where rapid access to exposure estimates could be beneficial once prospective models are established (**Figure** **4**). The concordance values (CCCs 0.733–0.895) and the proportion of predictions within 20–30% of serum concentrations observed here fall within the range reported in early evaluations of alternative sampling matrices such as saliva, dried blood spots, and interstitial fluid, supporting the technical feasibility of breath as an emerging medium for pharmacokinetic assessment.^19^, ^21^, ^22^

Several limitations should be considered. Interpretation of breath‐based exposure estimates should consider the concentration ranges represented in the present cohort. Model performance was evaluated primarily across therapeutic and subtherapeutic serum concentrations, with limited representation of extreme values and no systematic inclusion of toxic or overdose‐range concentrations for any ASM. As a result, prediction accuracy and error structure outside the observed concentration ranges cannot be inferred from the current data. Accordingly, the models should be interpreted as valid within the empirical bounds of the training data and not extrapolated beyond the concentration ranges evaluated here. Future studies incorporating broader concentration distributions enriched for supratherapeutic, toxic, and other clinically important out‐of‐range values will be required to characterize dynamic range, concentration‐dependent error, and model behavior at pharmacokinetic extremes more fully.

Sampling was not standardized to trough conditions, and variability in the interval between dosing and sample collection may have increased pharmacokinetic heterogeneity and affected concordance between breath‐derived and serum concentrations. The present findings therefore reflect performance under routine outpatient sampling conditions and should not be interpreted as validation for conventional trough‐based TDM. Polytherapy, including exposure to enzyme‐inducing and enzyme‐inhibiting ASMs, introduces biological variability that may influence breath‐derived metabolic signatures and affect model transportability across treatment regimens. Concomitant ASMs may also contribute overlapping drug‐derived or downstream endogenous metabolic signals, complicating attribution of individual features to a specific medication and potentially limiting generalizability to patients receiving monotherapy or different drug combinations. Although the overall cohort was relatively large for a breath‐based metabolomics study, subgroup sizes for individual ASMs were modest, limiting model precision as well as evaluation of negative predictive performance and clinical decision thresholds; larger cohorts enriched for out‐of‐range concentrations will be required to address these aspects.

Although exhalation flow, exhaled volume, end‐tidal CO_2_, room temperature, and transfer‐line temperature were monitored or standardized, residual variability related to respiratory physiology, airway conditions, compound volatilization, and analyte transfer cannot be excluded. The study also did not assess the analytical accuracy of individual metabolite measurements; its conclusions are therefore limited to prediction of systemic drug exposure from composite breath signatures rather than validated quantification of specific metabolites. While nested cross‐validation and Bayesian imputation strengthened internal validation, the single‐center design means that model transportability across settings remains uncertain; external multicenter validation across independent cohorts, instruments, and clinical environments is therefore essential to assess reproducibility and generalizability, particularly given the technical sensitivity of breath metabolomics to sampling methodology, humidity, instrument calibration, and instrument maintenance. Finally, online SESI‐HRMS currently requires proximity to a high‐resolution mass spectrometer, constraining immediate applicability in some clinical settings; however, ongoing developments in compact ionization sources, portable mass spectrometers, and offline breath‐collection approaches may broaden accessibility and enable breath sampling across a wider range of care environments prior to centralized analysis.^23^, ^24^, ^25^, ^26^

Compared with earlier studies focused predominantly on pediatric populations, individual drugs, or proof‐of‐principle detection, the present study provides a broader multi‐drug evaluation of breath‐based exposure estimation in adults with epilepsy. The retained predictive utility of the same 11‐feature VPA signature in the separate adult cohort supports its cross‐cohort transferability and biological plausibility within the present analytical framework, although targeted compound identification and confirmation across independent laboratories remain necessary. Extension to several newer ASMs further supports continued evaluation of breath metabolomics as a potential platform for multi‐drug exposure monitoring.

## CONCLUSIONS

This study supports the feasibility of non‐invasive breath‐based estimation of systemic exposure to multiple ASMs using SESI‐HRMS. Breath‐derived estimates showed moderate concordance with serum concentrations, but should currently be regarded as complementary screening information rather than substitutes for serum TDM. External multicenter validation across independent cohorts, instruments, and clinical environments is required before clinical implementation. Further validation may enable more accessible and scalable pharmacokinetic monitoring.

## FUNDING

This study was supported by the Lotte and Adolf Hotz‐Sprenger Foundation, the Schwyzer Foundation, the Uniscientia Foundation, and Lunge Zürich. PS received additional funding from the Foundation Botnar (Switzerland) and the Swiss National Science Foundation (grant numbers 320030_173168 and PCEGP3_181300). The funding bodies had no role in the design of the study; collection, analysis, and interpretation of data; or in writing the manuscript.

## CONFLICT OF INTEREST

MK reports consulting fees from Novartis, Roche Diagnostics, and GSK, and is co‐founder of Deep Breath Intelligence AG (Switzerland). MG reports receiving fees from Advisis, Angelini, Bial, Eisai, Neuraxpharm, and UCB outside the submitted work. PS is co‐founder and board observer of Deep Breath Intelligence AG. SU reports research grants from the Swiss National Science Foundation, Zurich Lung League, and EMDO Foundation, and has received grants, travel support, and consultancy fees from Orpha Swiss, Janssen SA, MSD SA, Gebro SA, and Ideogen, all outside the submitted work. KDS and FS are part‐time employees of Deep Breath Intelligence. All other authors declared no competing interests for this work.

## AUTHOR CONTRIBUTIONS

K.F. and D.M.B. wrote the manuscript; K.F., K.D.S., F.S., S.U., P.S., M.G., and M.K. designed the research; K.F., F.S., K.M., M.D.D., A.S., and J.H. performed the research; K.F., D.M.B., K.D.S., F.S., K.M., N.A.S., and P.S. analyzed the data.

## ETHICS APPROVAL AND CONSENT TO PARTICIPATE

The study was approved by the Cantonal Ethics Committee of Zurich (BASEC No. 2019‐00030) and conducted in accordance with the Declaration of Helsinki and Good Clinical Practice guidelines. All participants provided written informed consent prior to inclusion in the study.

## Supporting information


Tables S1–S2.

Table S3.

## Data Availability

The datasets generated and/or analyzed during the current study are included in this published article and the Supplementary Materials. Due to the inclusion of potentially identifiable human data, individual‐level clinical data are not publicly available but are available from the corresponding author on reasonable request and with permission of the Cantonal Ethics Committee of Zurich. The mass spectral features used for model development, including drug‐associated m/z features, are provided in Table S2 to support independent verification of the analytical approach. The data preprocessing and analysis pipeline is implemented in proprietary software (Deep Breath Intelligence AG, Switzerland) and is available through commercial licensing. All preprocessing steps, feature selection procedures, and model training workflows are described in sufficient detail in the Methods to enable independent replication.
