# Supplementary material for: Non‐Invasive Estimation of Systemic Drug Exposure Using Exhaled Breath Metabolomics in Humans: A Translational Proof‐of‐Concept Study
Source: Clin Pharmacol Ther. 2026 Aug 7:10.1002/cpt.70437. Online ahead of print. doi: 10.1002/cpt.70437 (PMC13451384; doi:10.1002/cpt.70437)
Supplement: Supplementary file 1 [file CPT-9999-0-s001.docx]

**Non-invasive estimation of systemic drug exposure using exhaled breath metabolomics: a human translational proof-of-concept study**

***Authors:*** *Kai Fricke, MD^1,2^; Diego M Baur, PhD^1,2^; Kapil Dev Singh, PhD^3,4^; Felix Schmidt, PhD^1,2^; Kathrin Müsch, PhD^1,2^; Noriane A Sievi, MSc^1,2^; Marco Di Donato, MD^2,5^; Anton Schmick, MD^2,5^; Jonas Herth, MD^1,2^; Silvia Ulrich, MD^1,2^; Pablo Sinues, PhD^3,4^; Marian Galovic, MD PhD^2,5^; Malcolm Kohler, MD^1,2^*

**Affiliations**:

1 Department of Pulmonology, University Hospital Zurich, Zurich, Switzerland

2 Faculty of Medicine, University of Zurich, Zurich, Switzerland

3 University Children’s Hospital Basel, Basel, Switzerland

4 Department of Biomedical Engineering, University of Basel, Basel, Switzerland

5 Department of Neurology, University Hospital Zurich, Zurich, Switzerland

***Table S1.* Reasons for study exclusion.**

| Study participant ID | Reason for Exclusion |
| --- | --- |
| PD_015 | No blood results available |
| PD_024 | Technical issues |
| PD_026 | No blood results available |
| PD_029 | Technical issues |
| PD_097 | Technical issues |
| PD_100 | Technical issues |
| PD_114 | No blood results available |

***Table S2:* Blood analysis methods for different antiseizure medications and their therapeutic window at University Hospital Zurich**

|  | Blood analysis | Therapeutic window | |
| --- | --- | --- | --- |
| Drug | Method | Reference range | Unit |
| Valproic acid, total | HEIA | 350-700 | µmol/l |
| Valproic acid, estimated free | Calculated^1^ | 35-69 | µmol/l |
| Levetiracetam | LC-MS/MS | 59-235 | µmol/l |
| Lamotrigine | LC-MS/MS | 11.7-58.6 | µmol/l |
| Lacosamide | LC-MS/MS | 1-10 | mg/l |
| ^1^ based on formula found in the following publication: Parent, X., Marzullo, C., & Gutbub, A. M. (1993, January). Valproic acid: a simple method for the estimation of free serum concentration. In *Annales de biologie clinique* (Vol. 51, No. 6, pp. 649-650). *Abbreviations:* HEIA = homogenous enzyme immunoassay; LC-MS/MS = liquid chromatography-tandem mass spectrometry; | | | |

***Table S4*. Exploratory classification performance relative to therapeutic reference intervals**

| ASM | Sample size (n) | Therapeutic range | Therapeutic range unit | PPV | NPV |
| --- | --- | --- | --- | --- | --- |
| Valproic acid (total) | 31 | 350-700 | µmol/L | 0.83 | 0.57 |
| Valproic acid (estimated free) | 28 | 35-69 | µmol/L | 0.84 | 0.57 |
| Levetiracetam | 34 | 59-235 | µmol/L | 0.85 | 0.57 |
| Lamotrigine | 72 | 11.7-58.6 | µmol/L | 0.84 | 0.33 |
| Lacosamide | 16 | 1-10 | mg/L | 1.00 | Not calculable |

This table summarises exploratory classification metrics for breath-based estimates of serum antiseizure medication concentrations relative to established therapeutic reference intervals. Positive predictive values and negative predictive values were calculated by categorising continuous model predictions as within or outside routinely used serum-based therapeutic ranges for each medication. These metrics are reported for completeness and comparative context only and should be interpreted in light of class imbalance, modest sample sizes for some medications, and limited representation of subtherapeutic or supratherapeutic concentrations within the study cohort. Negative predictive values could not be calculated for lacosamide because all measured serum concentrations for this medication fell within the therapeutic range. Estimated free valproic acid concentrations were derived using an albumin-based correction rather than direct measurement of unbound drug. Sample sizes reflect the number of paired measurements available for therapeutic classification after applying drug-specific inclusion criteria. Abbreviations: ASM = antiseizure medication; NPV = negative predictive value; PPV = positive predictive value;

**Supplementary Materials and Methods**

**Data Processing**

Raw mass spectra (Thermo Fisher .raw files) were pre-processed using a proprietary pipeline (Deep Breath Intelligence AG, Switzerland), implemented via in-house C# console applications built on Thermo Fisher Scientific’s RawFileReader and MATLAB. Following acquisition, end-tidal exhalation windows were defined from the Exhalion capnography trace (e.g., CO_2_ > 3%) to ensure that only the end-tidal fraction contributed to downstream analyses. Scans within exhalation windows were averaged to generate one representative averaged centroid spectrum per measurement followed by removal of satellite peaks and recalibration. Finally, the averaged centroid spectra from different measurements were binned within ± 2 ppm using MATLAB’s ksdensity function. In total, 19,790 mass spectral features were obtained (16,265 in positive ion mode and 3,525 in negative ion mode). Signal intensities were log₁₀-transformed. Features with more than 50% zero values were excluded. Remaining zero values were treated as left-censored and imputed using Bayesian lognormal modelling with Markov chain Monte Carlo sampling (PyMC v5.22.0; ArviZ v0.21.0). Model convergence was assessed using effective sample size and Gelman–Rubin statistics (R̂).

**Feature annotation and metabolite assignment**

Breath-derived molecular features were defined as reproducible m/z signals detected by SESI-HRMS in positive and negative ionisation modes. Putative metabolite annotations were assigned by accurate-mass matching against curated metabolite databases, taking into account elemental composition, ionisation mode, and known biochemical transformations. Metabolite identities should be interpreted as putative according to Metabolomics Standards Initiative level 2–3 (MSI criteria) rather than confirmed structures. Where multiple candidate annotations were possible for a given m/z feature, all plausible assignments were retained. Biological interpretation therefore focuses on consistency with known metabolic pathways rather than definitive identification of individual compounds.

**Machine Learning and Statistical Analysis**

Separate regression models were developed for each ASM (VPA, LEV, LTG, LCM) to predict serum drug concentrations from breath metabolomic data. Support vector machine regression (kernlab v0.9.33) and partial least squares regression (plsmod v1.0.0.9000, pls v2.8.5) were utilised for prediction.

Within each cross-validation fold, feature importance was evaluated using four complementary algorithms: least absolute shrinkage and selection operator (LASSO; glmnet v4.1.10), Boruta (Boruta v9.0.0), minimum redundancy maximum relevance (mRMR; mRMRe v2.1.2.2), and recursive feature elimination (RFE; caret v7.0.1). Feature importance scores were normalised and aggregated to generate a consensus ranking to improve robustness across feature selection methods.

Model training employed nested cross-validation, consisting of an outer 10-fold loop with 10 independent resamplings and an inner 10-fold cross-validation for hyperparameter tuning using tune_race_anova (finetune v1.2.0). To prevent information leakage, all measurements from the same participant were assigned to the same cross-validation fold. Model performance was summarised using Lin’s concordance correlation coefficient (CCC), root mean squared error (RMSE), mean absolute error (MAE), coefficient of determination (R²), and bias (yardstick v1.3.2). Samples lacking matched serum measurements were excluded from analysis.

**Threshold Calibration for Therapeutic Classification**

Continuous model predictions were categorised as within or outside the therapeutic range using established serum reference intervals: total VPA 350–700 µmol L⁻¹, free VPA 35–69 µmol L⁻¹, LEV 59–235 µmol L⁻¹, LCM 1–10 mg L⁻¹, and LTG 11.7–58.6 µmol L⁻¹. These thresholds correspond to accepted serum therapeutic ranges used in routine clinical TDM (**Table S2**). Classification performance was assessed using PPV, NPV, and AUC. These classification analyses are exploratory and dependent on cohort-specific distribution of concentrations.

**Model Interpretability and Uncertainty Quantification**

Feature importance rankings from LASSO, Boruta, mRMR, and RFE were combined to generate a consensus set of explanatory variables for final model interpretation. Model uncertainty was quantified using 1,000 bootstrap resamples. Median values and 95% confidence intervals were reported for CCC, AUC, PPV, NPV, RMSE, and MAE to assess robustness and reproducibility. All analyses were performed using R (v4.5.0; tidymodels v1.4.0, parsnip v1.4.0, recipes v1.3.1, rsample v1.3.1, workflowsets v1.1.1) and Python (v3.9.21).

**Reproducibility, transparency, and access to analytical workflows**

Feature alignment and peak matching were performed using fixed m/z tolerances defined a priori and did not depend on outcome variables or dataset-wide data distributions. All subsequent preprocessing steps, including feature filtering, normalisation, Bayesian imputation, and model training, were deterministic, parameter-fixed prior to analysis, and performed strictly within the training portion of each cross-validation fold before being applied unchanged to held-out test data, thereby minimising the risk of information leakage.

To support independent verification, the final lists of drug-associated m/z features used for model training are provided in **Table S3**. While the proprietary implementation is available through licensing, all preprocessing steps are fully specified to enable independent reproduction using alternative implementations.
